# Plasma Branched-Chain and Aromatic Amino Acids in Relation to Hypertension

**DOI:** 10.3390/nu12123791

**Published:** 2020-12-10

**Authors:** M. H. Mahbub, Natsu Yamaguchi, Ryosuke Hase, Hidekazu Takahashi, Yasutaka Ishimaru, Rie Watanabe, Hiroyuki Saito, Junki Shimokawa, Hiroshi Yamamoto, Shinya Kikuchi, Tsuyoshi Tanabe

**Affiliations:** 1 Department of Public Health and Preventive Medicine, Yamaguchi University Graduate School of Medicine, Ube, Yamaguchi 755-8505, Japan; natsu@yamaguchi-u.ac.jp (N.Y.); hase@umin.ac.jp (R.H.); ishimaru.yasutaka@pref.yamaguchi.lg.jp (Y.I.); rie-watanabe@kenwakai.gr.jp (R.W.); hirosaitojapan@gmail.com (H.S.); junki.shimokawa.och49@gmail.com (J.S.); tanabe@yamaguchi-u.ac.jp (T.T.); 2 Department of Public Health, Faculty of Veterinary Medicine, Okayama University of Science, Imabari, Ehime 794-8555, Japan; h-takahashi@vet.ous.ac.jp; 3 Institute for Innovation, Ajinomoto Co. Inc., Kawasaki, Kanagawa 210-8681, Japan; hiroshiA_yamamoto@ajinomoto.com (H.Y.); shinya_kikuchi@ajinomoto.com (S.K.)

**Keywords:** plasma amino acids, branched-chain, aromatic, hypertension

## Abstract

Findings of the available studies regarding the roles of branched-chain amino acids (BCAAs) and aromatic amino acids (AAAs) in hypertension are inconsistent, conflicting and inconclusive. The purpose of this study was to explore and clarify the existence of any relationships of individual BCAAs and AAAs with hypertension with adjustments for potential relevant confounders. A total of 2805 healthy controls and 2736 hypertensive patients were included in the current analysis. The associations between individual amino acids and hypertension were explored by logistic regression analyses adjusted for potential confounding variables. Among the investigated amino acids, only the BCAAs showed consistently significant positive associations with hypertension in the adjusted models (*p*-trend < 0.05 to 0.001). However, compared with the corresponding lowest quartile of individual BCAAs, the positive association with hypertension remained significant only in the highest quartile (*p* < 0.01 to 0.001). We confirmed in a relatively large cohort of subjects that BCAAs, not AAAs, demonstrated consistent positive associations with hypertension. The results display the promising potential for the use of BCAAs as relevant and accessible biomarkers, and provide perspectives on interventions directed towards the reduction in plasma BCAA levels in the prevention and management of hypertension.

## 1. Introduction

Currently, hypertension is the leading cause of global cardiovascular disease and premature deaths, and the leading single risk factor for the overall global burden of disease [1]. Worldwide, it affects more than 1.3 billion (31.1%) adults with an estimated overall prevalence of 31.5% in low- and middle-income countries (LMICs), and 28.5%, in high-income countries [2]. Moreover, due to the aging of the world population, the number of people with hypertension is expected to grow further as the incidence of it dramatically rises in line with increasing longevity [3]. Considering the extent and magnitude of this major public health problem, efforts directed towards better understandings and clarifications of the potential determinants and relevant underlying mechanisms are essential, which might help in early detection, effective prevention and/or better treatment of hypertension.

Under normal physiological conditions, individual amino acids play different metabolic or biochemical roles in the human body. On the other hand, under different pathological conditions, imbalances in circulating amino acids occur, which can exert specific negative effects on various physiological processes and organ functions [4]. Moreover, these analytes can be predictive of the development of a specific disease, serve as effective biomarkers for its detection and be highly responsive to therapeutic interventions [5,6]. Published literature suggests that individual amino acids can make independent and important contributions to the pathogenesis of hypertension [7,8]. Hence, revealing the potential association between specific circulating amino acids and hypertension might help to better understand the underlying disease pathophysiology, and undertake specific measures for the prevention and strategies for the management of hypertension.

In recent years, there has been a growing interest in investigating the potential effects of specific plasma free amino acids (PFAAs) on human blood pressure. Among the PFAAs, the association of branched-chain amino acids (BCAAs) (Isoleucine (Ile), Leucine (Leu), and Valine (Val)) and aromatic amino acids (AAAs) (Phenylalanine (Phe), Tyrosine (Tyr), and Tryptophane (Trp)) with hypertension has garnered particular attention. A number of recently published studies suggest that altered levels of BCAAs and AAAs are independently and significantly associated with hypertension [7,8,9]. However, the findings of the available clinical, epidemiological and experimental studies regarding the roles of BCAAs and AAAs in hypertension are inconsistent, conflicting and inconclusive, which show a positive association or no association of circulating concentrations of BCAAs and AAAs with hypertension [8,9,10,11,12]. Improved knowledge on the potential association between individual BCAAs and AAAs, and hypertension using a large study population, is of utmost importance as it might lead to the clarification and confirmation of the specific pattern of changes in BCAAs and/or AAAs in hypertension. Furthermore, it might help to better understand the underlying disease pathophysiology, undertake specific measures for the prevention of hypertension, and identify special biomarkers for the prevention and/or early detection and management of hypertension.

Considering the above-mentioned issues and controversies, one purpose of this study was to examine the differences in the concentrations of BCAAs and AAAs amongst apparently healthy control subjects versus hypertensive patients. Furthermore, we attempted to explore and clarify the existence of any relationships between the alterations in individual BCAAs and AAAs, and hypertension, with adjustments for potential relevant confounders.

## 2. Materials and Methods

### 2.1. Study Design and Ethical Issues

This cross-sectional study was conducted in Shimane Prefecture, Japan. The relevant institutional review boards of Shimane University (20100129-3) and Yamaguchi University (H25-26-2) approved the study protocol, which was conducted in accordance with the Declaration of Helsinki. The study participants were briefed verbally about the detailed protocol and provided written informed consent to participate in this study.

### 2.2. Study Population

**Figure 1:**
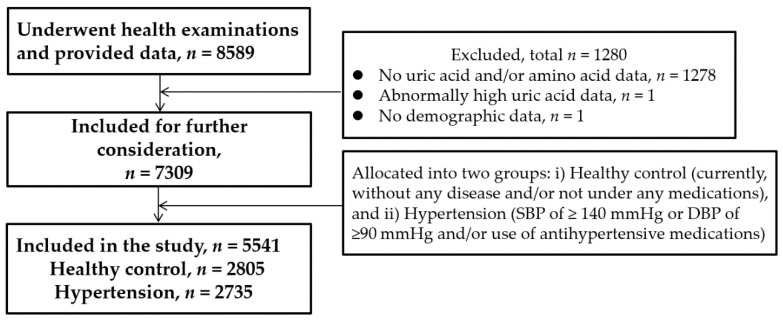
Flowchart of current study participants.

The procedures for selection of subjects have been described elsewhere [13,14]. Figure 1 depicts the flowchart of the participants included in this study. Briefly, the study population comprised 8589 subjects who underwent their annual health check-up that included physical examinations, clinical and other laboratory tests. In addition, data were collected on their personal and medical history using a self-administered questionnaire. For the current study, subjects were selected for the control (*n* = 2805) and hypertension (*n* = 2736) groups following the predefined criteria. The control group comprised apparently healthy subjects who were not under any medications. For inclusion in the second group, hypertension was defined as a systolic blood pressure (SBP) of ≥140 mmHg or a diastolic blood pressure (DBP) of ≥90 mmHg and/or the use of antihypertensive medication/s, according to the guidelines for the management of arterial hypertension from the European Society of Cardiology and the European Society of Hypertension [15]. Further exclusion of subjects with the coexistence of diabetes mellitus (defined as fasting plasma glucose (FPG) of ≥126 mg/dL or hemoglobin A1c/HbA1c of ≥6.5%), and/or the use of medication/s for diabetes mellitus [13] left 2736 subjects in the hypertension group for final inclusion in this study and analysis of the relevant data. The hypertensive subjects were free from any serious health problems such as cancer or renal failure.

### 2.3. Blood Sample Collection

During the check-up, venous blood samples were drawn from the cubital vein of a seated subject after an 8-h overnight fast. Briefly, 5 mL of blood samples were collected into tubes containing ethylenediaminetetraacetic acid (EDTA; Terumo, Tokyo, Japan), which were put on ice immediately and kept for about 15 min. Then, the tubes were incubated at 4 °C by centrifugation at 3000 rpm for 15 min. Subsequently, the plasma was separated into tubes, which were stored at −80 °C for a period 2 weeks to 2 months until the analysis for PFAAs was performed.

### 2.4. Measurements of Clinical and Laboratory Variables

On the examination day, measurements of resting arm blood pressures (systolic and diastolic) were performed with the subjects in sitting posture and their arm supported at heart level, by certified staff using automated noninvasive oscillometric arterial pressure measurement devices following the recommended guidelines for this purpose [16].

Following precolumn derivatization, the measurements of PFAA concentrations were performed by high-performance liquid chromatography–electrospray ionization–mass spectrometry (HPLC–ESI–MS), which allows such measurements with high accuracy. For this purpose, we followed the protocol as described elsewhere [17,18], and measured plasma levels of amino acids by using the so-called UF-Amino Station system (Shimadzu Corporation, Kyoto, Japan). The derivatization was performed with 3-aminopyridyl-N-hydroxysuccinimidylcarbamate (APDSTAG™, Wako Pure Chemical Industries, Ltd., Osaka, Japan). HPLC analysis was conducted on a Shim-pack UF-Amino column (C18 reverse-phase column, Shimadzu Corporation, Kyoto, Japan). Detection of the derivatized amino acids was achieved in ESI mode using a LCMS-2020 single quadrupole mass spectrometer. In this study, we determined the absolute concentrations (in μmol/L) of BCAAs and AAAs. Fasting plasma glucose (FPG) was measured by the hexokinase method. Plasma uric acid (UA) level was measured using the uricase-HMMPS method by L-type UA.M kit (Wako Pure Chemical Industries, Ltd., Osaka, Japan).

### 2.5. Statistical Analyses

The continuous variables of this study have been presented as the median and interquartile range (IQR). The differences for demographic and clinical variables between the two groups were assessed by the Mann–Whitney U-test for the continuous variables, and by the Chi-square (χ2) test for the categorical variables. Spearman’s rank correlation analysis was performed between the concentrations of BCAAs and AAAs separately in both groups. The patients with hypertension were categorized into four groups according to the quartile cut-off values determined using the distribution of each BCAA and AAA among the healthy control subjects, calculated separately for male and female subjects in both groups (Table S1).

In this study, we did not match the study populations and performed the multivariate tests to handle the confounding factors in the analyses [19]. At first, we investigated the crude association between all the individual BCAAs and AAAs with the outcome variable (hypertension) by the logistic regression analyses, as all these amino acids differed significantly between the two groups at *p* < 0.001. Next, the models were adjusted for the potential confounding demographic factors. Then, the logistic regression models were further adjusted with additional inclusion of SBP, DBP, FPG, UA, medication for hypertension, and medication for dyslipidemia as potential confounding factors. Trends were tested by treating PFAA quartiles as a continuous variable. From the logistic regression analyses, we obtained the odds ratios (OR) for individual amino acids with corresponding 95% confidence intervals (CI) and P-values. In addition, the P-values for tests of trends across quartiles of amino acids by the logistic regression analyses were obtained. The software package SPSS version 22 for Windows (SPSS Inc., Chicago, IL, USA) was used to perform the statistical analyses. All statistical tests were considered two-tailed, and a value of *p* < 0.05 was set as the significance level [20].

## 3. Results

A total of 2805 subjects (1192 men, 1613 women) in the healthy control group and 2736 subjects (1473 men, 1263 women) in the hypertension group were eligible for inclusion in the analysis of this study (Figure 1).

### 3.1. Demographic and Clinical Characteristics of Study Subjects

The demographic and clinical characteristics of the current study subjects have been presented in Table 1. Compared to the subjects in the healthy control group, the patients with hypertension were older with higher BMI and exhibited significantly higher values for systolic and diastolic blood pressure, FPG and UA (Mann–Whitney U-test, *p* < 0.001). In the latter group, 58.7% (1582/2697; missing, *n* = 39) and 23.9% (632/2646; missing, *n* = 90) of hypertensive patients were taking medications for hypertension and dyslipidemia, respectively. The control subjects did not take any medication (missing, *n* = 100).

### 3.2. Differences in the Concentrations of BCAAs and AAAs between Healthy and Hypertensive Subjects

Table 2 depicts the median and IQR values for the concentrations of individual BCAAs (Ile, Leu, and Val), and AAAs (Phe, Trp, and Tyr) in the healthy control and hypertension groups. For the BCAAs, the analyses revealed a significant increase in the concentrations of all individual PFAAs in the hypertension group when compared with those for the healthy control group (Mann–Whitney U-test, *p* < 0.001) (Table 2). Such a significant increase in the hypertension group was also observed for the AAAs (Mann–Whitney U-test, *p* < 0.001).

### 3.3. Correlation between BCAAs and AAAs

The relationships between measured concentrations of BCAAs and AAAs were examined with a correlation analysis and are presented in Table 3. As evident, the concentrations of BCAAs and AAAs showed significant positive correlations with moderate values in both healthy control (Spearman’s rank correlation, r = 0.42 to 0.58; *p* < 0.001) and hypertension (Spearman’s rank correlation, r = 0.44 to 0.53; *p* < 0.001) groups.

### 3.4. Association of BCAAs and AAAs with Hypertension

The associations of the concentration of BCAAs and AAAs with hypertension (no versus yes) were evaluated with crude and adjusted logistic regression models. In the crude model, all BCAAs and AAAs showed significant positive associations with hypertension (*p*-trend < 0.001). Analyses by logistic regression also revealed that all PFAAs had a linear relationship across all the quartile categories. Overall, the significant associations with hypertension were stronger in the higher quartile categories when compared with those for the corresponding lowest quartile category of individual PFAAs (OR between 1.19 and 2.83, 95% CI between 1.01 and 2.43 (lower) and 1.40 and 3.30 (upper), *p* < 0.05 to 0.001 for BCAAs; OR between 1.46 and 5.23, 95% CI between 1.26 and 4.42 (lower) and 1.69 and 6.19 (upper), *p* < 0.001 for AAAs) (Table 4).

In contrast, in both adjusted models, only the BCAAs demonstrated consistently significant positive associations with hypertension (*p*-trend < 0.05 to 0.001). However, in these models, compared with the corresponding lowest quartile category of individual BCAAs, the positive association with hypertension remained significant only in the highest quartile category. In the highest quartile category of the final model, the ORs were 2.30, 2.41, and 2.19 with 95% CIs of 1.31–4.04, 1.35–4.33 and 1.25–3.85 for Ile, Leu and Val, respectively; all *p* < 0.05.

Among the AAAs, only Tyr showed a significant trend of association with hypertension in the second model adjusted for age, sex and BMI (*p*-trend < 0.05). When compared with the lowest quartile category, the association was significant in the highest quartile category of Tyr. On the other hand, although Trp demonstrated significant associations in the upper two quartile categories of the final model in comparison with the lowest quartile category (*p* < 0.05), the trend of that association did not reach statistical significance (*p*-trend = 0.053).

## 4. Discussion

Alterations in the circulating levels of BCAAs and AAAs have a profound effect on cell signaling, gene expression and neuroendocrine function, and might be associated with hypertension [21,22,23]. In light of the existing conflicting reports on the association between alterations in the plasma levels of BCAAs and AAAs with hypertension, in this study, we characterized and confirmed the potential existence of distinct patterns of associations of these PFAAs with the latter.

As reported in the literature, the prevalence of hypertension progressively increases with age and there is a positive association of increased risk of hypertension with a higher BMI [24,25]. Therefore, our findings of significantly higher values of age and BMI among the subjects with hypertension are in line with the existing literature.

Existing evidence also indicates a clear relationship of an elevated level of circulating UA (known as hyperuricemia) with hypertension. Furthermore, it has been mentioned that the role of hyperuricemia might be causal in the development of hypertension [26]. Therefore, our finding of a significantly higher concentration of UA among the subjects with hypertension is in line with the current evidence. Considering such a role of elevated level UA in hypertension, we adjusted our results of logistic regression analysis for UA along with other investigated variables. In addition, we adjusted the results of logistic regression analysis for FPG as a strong relationship between the plasma level of glucose and amino acids has been described in the literature [5,20].

In our study, compared to the healthy controls, the concentrations of all BCAAs and AAAs showed significant elevations amongst the subjects with hypertension. Our findings correspond to those of a previously published study that was conducted among a separate Japanese population and compared the baseline values of PFAAs among subjects including those with and without hypertension [20]. In this study, the crude associations of all individual BCAAs and AAAs with hypertension were significant. It has been suggested that BCAAs and AAAs have hydrophobic or bulky residues that can be relevant for the binding of bioactive peptides to the angiotensin-converting enzyme which is crucial in blood pressure control [27]. There is a possibility that the increased levels of circulating BCAAs and AAAs can enhance the angiotensin converting enzyme activity through increased binding of these amino acid residues to the latter leading to a rise in blood pressure.

A number of recent epidemiological studies investigated the association of BCAAs and AAAs with hypertension; but the published data show discrepant and inconsistent results. For example, Yang et al. [12] observed significant positive correlations between individual serum BCAAs with DBP, and only serum Val with SBP. Similarly, Siomkajło et al. [28] found significant positive associations between plasma BCAAs, and systolic and diastolic blood pressure. In addition, in a large-scale prospective cohort study, high concentrations of BCAAs were associated with an incident hypertension in middle-aged men and women [9]. In contrast, BCAA levels were not correlated with systolic and diastolic blood pressures in a cross-sectional study conducted among a Chinese population [10]. On the other hand, the adjusted logistic regression models in our study demonstrated a significant trend of positive associations between BCAAs and hypertension with stronger associations in the higher quartile categories of all BCAAs. Our findings raise the possibility of the probable relationship between altered levels of BCAAs with the severity of hypertension.

The exact mechanisms underlying the association between all individual BCAAs and hypertension have not been clarified and/or established yet. BCAAs function not only as important building blocks for tissue protein, but also as important signaling molecules and cell regulators influencing key cell signaling pathways [29]. Yang et al. [30] postulated that among the BCAAs, Leu may act as an inhibitor of NO synthesis from L-arginine in endothelial cells. In addition, as mentioned in the literature, Leu may induce an increase in blood pressure as its transamination can promote the formation of glutamate with subsequent generation of alanine, the urinary excretion of which demonstrated a strong association with higher blood pressure amongst the participants in the INTERMAP epidemiological study [31,32]. Moreover, it has been suggested that that the alterations in BCAA metabolism with accumulation of BCAAs and their byproducts can cause remarkable metabolic derangements with impairments in the function of the mammalian target of rapamycin complex 1 (mTORC1), and overstimulation of adenosine monophosphate activated protein kinase (AMPK) and general control nonderepressible 2 (GCN2), which subsequently leads to insulin resistance and oxidative stress playing key roles in the regulation of blood pressure and can cause the development of hypertension [33,34,35].

In this study, the results of adjusted logistic regression models could not reveal any consistent trend in associations between AAAs and hypertension, although the concentrations of AAAs were significantly higher among the subjects with hypertension compared with the control subjects. This might reflect the existence of a possible close link between the altered levels of BCAAs and AAAs, and is in line with significant positive correlations observed between these two types of PFAAs in this study. In human blood, approximately 40% of the free essential amino acids are comprised of BCAAs. It has been suggested that BCAAs and AAAs compete for transport into mammalian cells by the common large neutral amino acid transporter (LAT1) [36,37]. Therefore, a significant increase in the AAA levels among the hypertensive subjects might have been partially driven by the higher levels of BCAAs among them.

In our study, among the AAAs, Tyr level in the highest quartile category showed a significant positive association with hypertension after adjustments for age, sex and BMI (model 2). On the other hand, Trp level in the upper quartile categories retained the significant association with hypertension in the fully adjusted logistic regression model (model 3). It has been suggested that among the AAAs, the effects on blood pressure are potentially due to the changes in the levels of Tyr as it acts as a precursor for norepinephrine synthesis, and thereby can modulate norepinephrine levels and affect the sympathetic vascular tone [8]. Furthermore, the majority of Phe is converted to Tyr, potentiating the effect of the latter on the vascular tone [8]. In contrast, in an animal experiment, there had been a six-fold rise in the brain concentration of Trp in chronic hypertensive rats, as compared to normotensive rats [38]. Watts et al. [39] suggested that Trp, being the precursor for the synthesis of serotonin, might potentially influence the vascular tone through the serotonin receptors present on adrenergic nerves at the level of the sympathetic vasculature. In a Polish observational study assessing the plasma amino acid levels, statistically significant correlations with SBP and DBP were identified by the principal component analysis for a cluster of PFAAs that included Phe besides BCAAs [28]. On the other hand, Yang et al. [12] observed significant positive correlations between each individual serum AAAs with DBP. All the findings and observations mentioned above demonstrate a profound lack of consensus among the researchers regarding the association of circulating AAAs with hypertension. Therefore, considering our findings and those of other studies, we postulate that the relationship of AAAs with hypertension is uncertain. On the other hand, the relevant findings indicate the existence of a clear positive association between BCAAs and hypertension.

There are several potential limitations to this study and caution is required while interpreting the current results. First, the generalization of our study findings is somewhat limited as this study was conducted amongst the Japanese population. The observed associations between BCAAs and AAAs, and hypertension, may be different in other populations, especially in the younger people, which deserves further investigation. Second, data on background information such as diet, physical exercise, smoking status or alcohol consumption, etc., were not available for this study. However, we believe that any such effects on the current results should be limited as we included a separate control group without hypertension, but with similar socio-demographic characteristics. Another potential limitation might be the fact that we did not stratify our results by sex. We believe this does not influence our study findings as we used different criteria for men and women while classifying the data of each PFAA according to their quartiles. Moreover, we have presented the results of logistic regression analyses after adjustments for a set of potential confounders including the variable “sex”. The cross-sectional nature of our study design does not allow us to speculate on any causality or temporality for any associations observed by us. In addition, our study design did not allow us to verify any pathophysiological mechanism underlying the relationships between altered levels of PFAAs and hypertension. Lastly, our observed associations between circulating BCAAs and hypertension should be interpreted with care as the metabolism of BCAAs is remarkably complex, and the plasma levels of BCAAs do not necessarily correspond to their intracellular levels in different types of cells.

## 5. Conclusions

Our study provides evidence for the potential existence of a close relationship between high concentrations of BCAAs with hypertension, after adjustment for possible confounding factors. The current findings might help to increase our understanding on the role of altered levels of BCAAs in hypertension, and offer new opportunities in the prevention of this important disease. In the future, longitudinal studies should investigate the possibility of using circulating BCAAs as novel biomarkers for evaluating the risk of incident hypertension, and also for early detection and monitoring of hypertension. Furthermore, it would be necessary to investigate the effectiveness of any interventions or manipulations directed towards the reduction in plasma BCAA levels, which would be valuable in developing potential new strategies for the prevention and management of hypertension.

## Figures and Tables

**Table 1:** Demographic and clinical characteristics of study subjects. Values are expressed as median and interquartile range (IQR) for continuous variables, and number and percent for the categorical variable.

Characteristics	Healthy		Hypertension		*p*-Value
	(*n* = 2805)		(*n* = 2736)		
	Median or *n*	IQR or %	Median or *n*	IQR or %	
Age (years)	43	25	64	18	<0.001
Sex					<0.001
Male	1192	42.5	1473	53.8	
Female	1613	57.5	1263	46.2	
BMI (kg/m^2^)	21.3	3.7	23.6	4.2	<0.001
SBP (mmHg)	118	15	140	17	<0.001
DBP (mmHg)	73	13	86	15	<0.001
FPG (mg/dL)	91	10	98	14	<0.001
UA (mg/dL)	4.6	1.9	5.3	2	<0.001

BMI, body mass index; SBP, systolic blood pressure; DBP, diastolic blood pressure; FPG, fasting plasma glucose; UA, uric acid.

**Table 2:** Branched-chain amino acid (BCAA) and aromatic amino acid (AAA) concentrations (μmol/L) in the study populations. Values are shown as median and interquartile range (IQR).

Amino Acids	Healthy		Hypertension		*p*-Value
	(*n* = 2805)		(*n* = 2736)		
	Median	IQR	Median	IQR	
Ile	51.5	16.0	57.4	18.7	<0.001
Leu	102.6	29.3	112.7	33.3	<0.001
Val	190.8	51.0	211.8	54.8	<0.001
Phe	53.7	10.2	58.9	10.8	<0.001
Tyr	57.2	14.8	65.4	17.0	<0.001
Trp	51.2	11.9	53.7	12.0	<0.001

Ile, isoleucine; Leu, leucine; Val, valine; Phe, phenylalanine; Trp, tryptophan; Tyr, tyrosine. P-values indicate the differences by two-tailed Mann–Whitney U-test between healthy control and hypertension groups.

**Table 3:** Correlation coefficients between the concentrations of branched-chain amino acids (BCAAs) and aromatic amino acids (AAAs) for healthy control subjects and subjects with hypertension derived by Spearman’s rank correlation analysis.

	Healthy: AAAs			Hypertension: AAAs		
BCAAs	Phe	Tyr	Trp	Phe	Tyr	Trp
Ile	0.48 *	0.42 *	0.52 *	0.48 *	0.45 *	0.49 *
Leu	0.58 *	0.47 *	0.56 *	0.53 *	0.46 *	0.53 *
Val	0.52 *	0.45 *	0.55 *	0.47 *	0.44 *	0.49 *

* All *p*-values: <0.001. Ile, isoleucine; Leu, leucine; Val, valine; Phe, phenylalanine; Tyr, tyrosine; Trp, tryptophan.

**Table 4:** Logistic regression analysis for association between hypertension with quartiles of individual branched-chain and aromatic amino acids without and with adjustments for potential confounding factors.

Amino Acids	Quartile Categories	Model 1 ^a^					Model 2 ^b^					Model 3 ^c^				
		OR	95% CI			*p*-Value	OR	95% CI			*p*-Value	OR	95% CI			*p*-Value
			Lower	Upper	*p*-Value	(Trend) ^d^		Lower	Upper	*p*-Value	(Trend) ^d^		Lower	Upper	*p*-Value	(Trend) ^d^
Ile	1	Ref	–	–	–		Ref	–	–	–		Ref	–	–	–	
	2	1.10	0.93	1.30	0.257	<0.001	1.01	0.82	1.23	0.952	<0.001	1.11	0.62	2.02	0.721	<0.05
	3	1.46	1.25	1.71	<0.001		1.21	1.00	1.48	0.056		1.63	0.93	2.87	0.091	
	4	2.43	2.08	2.82	<0.001		1.65	1.36	1.99	<0.001		2.30	1.31	4.04	<0.005	
Leu	1	Ref	–	–	–		Ref	–	–	–		Ref	–	–	–	
	2	1.19	1.01	1.40	0.041	<0.001	1.03	0.84	1.26	0.799	<0.001	1.78	0.97	3.24	0.063	<0.05
	3	1.34	1.14	1.57	<0.001		1.14	0.93	1.39	0.213		1.48	0.83	2.62	0.185	
	4	2.47	2.13	2.88	<0.001		1.49	1.23	1.81	<0.001		2.41	1.35	4.33	<0.005	
Val	1	Ref	–	–	–		Ref	–	–	–		Ref	–	–	–	
	2	1.19	1.00	1.40	0.050	<0.001	1.02	0.83	1.25	0.886	<0.001	1.22	0.68	2.20	0.508	<0.05
	3	1.47	1.25	1.73	<0.001		1.11	0.90	1.36	0.327		1.50	0.82	2.74	0.185	
	4	2.83	2.43	3.30	<0.001		1.53	1.26	1.86	<0.001		2.19	1.25	3.85	<0.01	
Phe	1	Ref	–	–	–		Ref	–	–	–		Ref	–	–	–	
	2	1.55	1.30	1.85	<0.001	<0.001	0.89	0.72	1.11	0.310	0.355	1.34	0.72	2.49	0.359	0.654
	3	2.26	1.90	2.67	<0.001		0.89	0.72	1.10	0.264		1.49	0.79	2.78	0.217	
	4	4.23	3.59	4.98	<0.001		1.01	0.82	1.24	0.966		1.40	0.75	2.63	0.291	
Tyr	1	Ref	–	–	–		Ref	–	–	–		Ref	–	–	–	
	2	1.62	1.35	1.95	<0.001	<0.001	0.99	0.79	1.24	0.896	0.026	1.12	0.56	2.27	0.747	0.601
	3	2.60	2.18	3.10	<0.001		1.11	0.89	1.37	0.365		1.48	0.79	2.79	0.223	
	4	5.22	4.41	6.18	<0.001		1.27	1.03	1.57	0.025		1.37	0.72	2.62	0.336	
Trp	1	Ref	–	–	–		Ref	–	–	–		Ref	–	–	–	
	2	0.95	0.81	1.11	0.493	<0.001	0.96	0.79	1.16	0.652	0.153	1.63	0.91	2.93	0.102	0.053
	3	1.15	0.98	1.33	0.081		1.10	0.91	1.33	0.321		2.10	1.20	3.70	<0.01	
	4	1.45	1.25	1.68	<0.001		1.16	0.97	1.40	0.111		1.94	1.10	3.40	<0.05	

^a^ Without adjustment; ^b^ adjusted for age, sex and BMI; ^c^ adjusted for age, sex, BMI, SBP, DBP, FPG, UA, medication for hypertension and medication for dyslipidemia. ^d^ Test of trend across quartiles of amino acids by logistic regression analysis. Ile, isoleucine; Leu, leucine; Val, valine; Phe, phenylalanine; Tyr, tyrosine; Trp, tryptophan.

## Supplementary Materials

The following is available online at https://www.mdpi.com/2072-6643/12/12/3791/s1, Table S1: Quartile cut-off values of BCAAs and AAAs, and the corresponding distribution of subjects across quartile categories.
