# Supplementary material for: Plasma Branched-Chain and Aromatic Amino Acids in Relation to Hypertension
Source: Nutrients. 2020 Dec 10;12(12):3791. doi: 10.3390/nu12123791 (PMC7764357; doi:10.3390/nu12123791)
Supplement: Supplementary file 1 [file nutrients-12-03791-s001.pdf]

nutrients

Supplementary Material

Table S1. Quartile cut-off values (µmol/L) of branched-chain amino acids (BCAAs) and aromatic amino acids (AAAs), and the corresponding distribution of study subjects across quartile categories.

Amino.acids	Quartile categories	Cut-off values		Healthy		Hypertension	
		Male	Female	Male, *n*	Female, *n*	Male, *n*	Female, *n*
**Ile**	1	<53.55	<41.42	298	403	267	190
	2	53.55 to <60.54	41.42 to <46.10	298	405	299	205
	3	60.54 to <68.86	46.10 to <51.91	298	402	354	313
	4	≥68.86	≥51.91	298	403	553	555
Leu	1	<107.62	<83.16	298	403	292	164
	2	107.62 to <119.43	83.16 to <93.10	298	404	273	269
	3	119.43 to <131.66	93.10 to <102.52	298	403	329	281
	4	≥131.66	≥102.52	298	403	579	549
Val	1	<196.33	<157.25	298	403	272	150
	2	196.33 to <216.30	157.25 to <174.99	298	404	288	213
	3	216.30 to <238.97	174.99 to <193.15	298	403	356	263
	4	≥238.97	≥193.15	298	403	557	637
Phe	1	<52.71	<47.39	298	403	180	123
	2	52.71 to <57.35	47.39 to <51.44	298	404	274	196
	3	57.35 to <62.79	51.44 to <55.80	298	403	390	292
	4	≥62.79	≥55.80	298	403	629	652
Tyr	1	<54.61	<48.31	298	403	159	103
	2	54.61to <61.44	48.31 to <54.28	298	404	236	189
	3	61.44 to <69.17	54.28 to <61.01	298	403	371	310
	4	≥69.17	≥61.01	298	403	707	661
Trp	1	<51.69	<43.14	298	403	367	235
	2	51.69 to <56.91	43.14 to <47.78	298	404	330	241
	3	56.91 to <62.36	47.78 to <52.32	298	403	363	326
	4	≥62.36	≥52.32	298	403	413	461

Ile, isoleucine; Leu, leucine; Lys, lysine; Phe, phenylalanine; Trp, tryptophan; Tyr, tyrosine; Val, valine.

© 2020 by the authors. Submitted for possible open access publication under the terms and conditions of the Creative Commons Attribution (CC BY) license (http://creativecommons.org/licenses/by/4.0/).
